# Baicalein Accelerates Tendon-Bone Healing via Activation of Wnt/*β*-Catenin Signaling Pathway in Rats

**DOI:** 10.1155/2018/3849760

**Published:** 2018-03-06

**Authors:** Xinggui Tian, Huaji Jiang, Yuhui Chen, Xiang Ao, Chuan Chen, Wentao Zhang, Feilin He, Xiaoqing Liao, Xiaocheng Jiang, Tao Li, Zhongmin Zhang, Xintao Zhang

**Affiliations:** ^1^Department of Orthopedics, The Third Affiliated Hospital of Southern Medical University, Guangzhou 510515, China; ^2^Department of Spine Surgery, The Affiliated Hospital of Southwest Medical University, Luzhou, Sichuan 646000, China; ^3^Department of Pain, Yue Bei People's Hospital, Shaoguan, Guangdong 512000, China; ^4^Department of Orthopedics, The First People's Hospital of Guangyuan, Guangyuan, Sichuan 628017, China; ^5^Department of Sports Medicine and Rehabilitation, Peking University Shenzhen Hospital, Shenzhen 518000, China

## Abstract

**Background:**

Tendon-bone healing is a reconstructive procedure which requires a tendon graft healing to a bone tunnel or to the surface of bone after the junction injury between tendon, ligament, and bone. The surgical reattachment of tendon to bone often fails due to regeneration failure of the specialized tendon-bone junction.

**Materials and Methods:**

An extra-articular tendon-bone healing rat model was established to discuss the effect of the baicalein 10 mg/(kg·d) in accelerating tendon-bone healing progress. Also, tendon-derived stem cells (TDSCs) were treated with various concentrations of baicalein or dickkopf-1 (DKK-1) to stimulate differentiation for 14 days.

**Results:**

* In vivo,* tendon-bone healing strength of experiment group was obviously stronger than the control group in 3 weeks as well as in 6 weeks. And there were more mature fibroblasts, more Sharpey fibers, and larger new bone formation area treated intragastrically with baicalein compared with rats that were treated with vehicle for 3 weeks and 6 weeks.* In vitro,* after induction for 14 days, the expressions of osteoblast differentiation markers, that is, alkaline phosphatase (ALP), runt-related transcription factor 2 (Runx2), osteocalcin (OCN), osterix (OSX), and collagen I, were upregulated and Wnt/*β*-catenin signaling pathway was enhanced in TDSCs. The effect of DKK-1 significantly reduced the effect of baicalein on the osteogenic differentiation.

**Conclusion:**

These data suggest that baicalein may stimulate TDSCs osteogenic differentiation via activation of Wnt/*β*-catenin signaling pathway to accelerate tendon-bone healing.

## 1. Background

Tendon-bone healing is a reconstructive procedure which requires a tendon graft healing to a bone tunnel (e.g., anterior cruciate ligament reconstruction) or to the surface of bone (e.g., rotator cuff tendon repair) after the junction injury between tendon, ligament, and bone. Anterior cruciate ligament reconstruction is the most common tendon-bone healing surgery, and successful reconstruction requires the tendon graft effective healing in the bone tunnels [1, 2]. The surgical reattachment of tendon to bone often fails due to regeneration failure of the specialized tendon-bone junction [3]. In the early healing period, it is essential to delay activities to protect the graft from overloading, because the attachment between tendon graft and bone is weak. Improvement in graft healing to bone is critical to allow earlier rehabilitation and work or other special activities [1, 2]. Some studies have demonstrated that the reason of poor tendon-bone healing result is that tendon graft in a bone tunnel osseous ingrowth, mineralization, and maturation is not always satisfactory. So, by stimulating osteointegration of the healing tendon graft can accelerate the tendon-bone healing progress [1, 2, 4, 5].

Currently, some studies have demonstrated that many biological agents can speed up the healing rate and improve the quality of healing, such as artificial composite bone [6], periosteum [2], biodegradable interference screw [4], calcium bone cement [7], growth factors [8–10], bone morphogenetic protein [1, 11–13], and stem cells [14–16]. However, those clinical uses are still limited because of possible adverse effects of permanent implants and difficulty determining the appropriate dose in surgery [1, 2]. At the same time, some agents are expensive and difficult to obtain, which are difficult to be popularized in clinical use. Consequently, it is an urgent task to identify some biological agents that have better and safer effect to treat tendon-bone healing.

Baicalein is the main active components of* Scutellaria baicalensis* which is one of the Chinese herbs commonly used in products prescribed for the treatment of bone and joint diseases for thousands of years. Past studies have reported that baicalein can stimulate osteoblast differentiation [17, 18] and inhibit osteoclast differentiation [19]. Therefore, baicalein may have the possibility of enhancing tendon-bone healing through promoting osteoblast differentiation of the tendon graft in a bone tunnel.

Tendon-derived stem cells (TDSCs) have been identified within tendon tissues by Bi et al. (2007), which have universal stem cell characteristics such as clonogenicity, multipotency, and self-renewal capacity [20]. TDSCs can differentiate into adipocytes, chondrocytes, and osteocytes* in vitro* and formed tendon-like, cartilage-like, bone-like, and tendon–bone junction-like tissues in rat models [20, 21]. The main potential benefit of TDSCs is that they are resident cells in the tendon, so using TDSCs as the cell source for tendon-bone junction repair has more advantages than other mesenchymal stem cells [21, 22]. The TDSCs can be triggered by the inflammatory reaction after tendon injury in rats [23]. Some studies have shown that TDSCs are capable of promoting tendon and tendon-to-bone healing [22, 24–26]. But the underlying molecular mechanisms of TDSCs promoting tendon-bone healing remain poorly understood. Therefore, TDSCs may be a potential target for treatment in tendon-bone healing.

In this study, we tested the effect of baicalein on rat tendon-bone healing, discussed the effect of baicalein on TDSCs proliferation and osteogenic differentiation, and clarified its related mechanisms of baicalein in TDSCs. Also, we tested the hypothesis that baicalein accelerates tendon-bone healing by stimulating osteogenic differentiation of TDSCs. The results demonstrate that baicalein induces TDSCs osteogenic differentiation by activation of Wnt/*β*-catenin signaling pathway to accelerate tendon-bone healing progress.

## 2. Materials and Methods

### 2.1. Reagents

Baicalein was purchased from Aladdin (product number B107323, Shanghai Aladdin Biochemical Technology Co., Ltd., Shanghai, China). According to baicalein's product specification, we can get that its formula weight is 270.24 g/M and its purity is no less than 98%. We dissolved the baicalein in phosphate-buffered saline (PBS, Shanghai Sangon Biotech Co., Ltd., Shanghai, China) to make stock solutions and stored them at −20°C.

### 2.2. Animal Model and Drug Treatment

**Figure 1:**
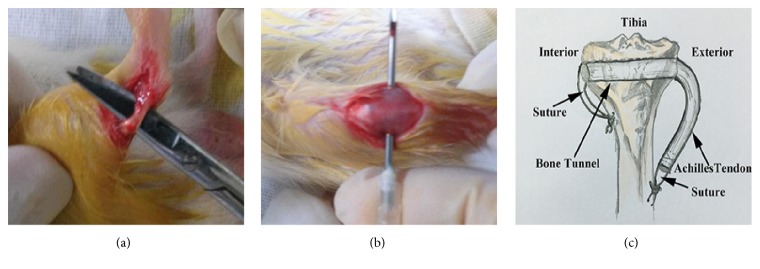
Surgery procedure of tendon-bone healing tunnel mode. (a) Tendon isolation; (b) bone tunnel creation; (c) tendon penetrated bone tunnel and suture for fixing tendon.

Forty Sprague-Dawley male rats (180–220 g, purchased from the center of experimental animals of Southern Medical University) which were randomly divided into 2 groups each were anaesthetised with an intraperitoneal injection 10% chloral hydrate (0.4 ml/100 g, Beijing Solarbio Science & Technology Co., Ltd., Beijing, China) to induce general anesthesia. The rats were operated on their right hind limb to build extra-articular tendon-bone healing model. A 10 mm longitudinal skin incision was made on the medial side of the right Achilles tendon and the fascia cut longitudinally. The Achilles tendon was released from calcaneal tuberosity to calf muscle (Figure 1(a)). And then a 10 mm midline knee incision was made. A tunnel was drilled in the proximal tibial metaphysis with a needle of 50 ml syringe perpendicular to the long axis of the tibia (Figure 1(b)). The graft was pulled manually into the bone tunnel. The ends of grafts were sutured into the adjacent periosteum and soft tissue and wound were closed in layers (Figure 1(c)) [27]. The experiment group was treated intragastrically with baicalein (10 mg/kg/d) for 3 weeks or 6 weeks, while the control group was treated with normal saline. After euthanasia, tendon graft-tibia tunnel complex was dissected and fixed in 4% paraformaldehyde (Sigma-Aldrich, St Louis, MO, USA) at 3 and 6 weeks after surgery. The degree of tendon-bone healing was evaluated with biomechanical and histological testing. Animal treatment conformed to the animal care guidelines of Southern Medical University laboratory animal welfare and ethics committee charter.

### 2.3. Biomechanical Testing

At each time point, five tendon graft-tibia tunnel complex samples were used for biomechanical testing. The tibia was removed from knee joint to ankle joint and the fibula was removed. All muscles and periosteum around complex and the fixed sutures at the ends of tendon were carefully removed, but the tendon graft with surrounding callus tissue was left undamaged. The specimens were stored at 20°C and were placed at room temperature for 3 h before the testing. The free end of tendon and tibia tunnel were mounted in the testing jigs of the material testing device before load. The tensile stress was consistent with the direction of the bone tunnel during the testing at a constant speed of 6 mm/min, and the sample was kept moist with normal saline. The pull-out strength (N) was defined as the maximum load at which the tendon was pulled out or failure load at which the tendon was ruptured, and the data were generated automatically by the computer software system (Laboratory, Technology, Corporation, USA). The length of the bone tunnel (mm) was tested by a vernier caliper. Tendon-bone healing strength (N/mm) was expressed by the ratio of maximum load or failure load (N) to the length of the bone tunnel (mm).

### 2.4. Haematoxylin and Eosin (H&E) Staining

The samples were decalcified using ethylenediaminetetraacetic acid (EDTA, Guangzhou Chemical Reagent Factory, Guangzhou, China) for 1 month. And then the samples were embedded in paraffin (bone tunnel perpendicular to paraffin surface) and 5 um thick sections were cut. The serial sectioning method obtained the different parts of the tissue sections of the tunnel. The sections were stained with haematoxylin and eosin (H&E) and considered by microscopy (Axio Scope A1, Carl Zeiss Microscopy GmbH, Jena, Germany).

### 2.5. Cell Culture

Ten healthy male Sprague-Dawley rats (6 weeks old) were used to get TDSCs. Cells cultured in complete medium consisting of Dulbecco's modified Eagle's medium (DMEM, Hyclone, Guangzhou, China), 1% penicillin/streptomycin (Beyotime Biotechnology Co. Ltd., Shanghai, China), and 10% fetal bovine serum (FBS, Gibco, Carlsbad, CA, USA). The cells were seeded in 10-cm dishes in a humidified 5% CO_2_ atmosphere at 37°C. The medium of cells was replaced every 3 days, and nonadherent cells were removed [28, 29]. Cells at passage 3 were used only for following experiments.

### 2.6. CCK-8 Experiment

According to the instructions of cell counting kit-8 (CCK-8; Dojindo Molecular Technologies; Japan) colorimetric assay, we used it to detect cell proliferation and viability. TDSCs were seeded into 96-well plates at a density of 5 × 10^4^ cells/well and incubated 37°C, 5% CO_2_ incubator, for 24 hours; each group was mixed with 0.1, 1, 10, 100, and 1000 uM baicalein, respectively. 14 days later, CCK-8 Kit was added to each well and then incubated for 4 hours. The optical density values were checked at 450 nm by a multifunctional microplate reader (Synergy HTX, Bio-Tek Instruments, Inc., Winooski, VT, USA) to test cell proliferation and viability index.

### 2.7. ALP Activity/Staining Assay

TDSCs were digested with 0.02% EDTA and trypsin 1 : 1 mixture (Guangzhou Whiga Technology Co., Ltd., Guangzhou, China), inoculated in 6-well plates at a density of 2 × 10^5^ cells/well, and cultured with the osteoblast differentiation medium (DMEM, 10% FBS, 1% penicillin, and streptomycin, 10 mM *β*-phosphate glycerol, 0.1 uM dexamethasone, 50 uM L-ascorbic acid) containing appropriate concentrations (0-10 uM) baicalein. The medium culturing cells were replaced every 3 days. Two weeks later, the medium of 6-well plates culturing cells was removed and the cells were washed twice in PBS. And then TDSCs were lysed with 0.5 ml of 0.1% T-octylphenoxypolyethoxyethanol X-100 (TritonX-100, Shanghai Sangon Biotech Co., Ltd., Shanghai, China) at room temperature for 15 minutes. ALP activity was checked using a fluorometric detection kit according to the instructions (Nanjing Jiancheng Biotechnology Co., Ltd., Jiangsu, China). According to the instructions of ALP staining (Beyotime Biotechnology), parallel wells were added to ALP staining buffer to observe the quantity of the ALP. The results were observed by taking photos with a microscope (Olympus Corporation, Tokyo, Japan).

### 2.8. Alizarin Red Staining and Mineralized Nodules

The TDSCs were inoculated at a density of 2 × 10^5^ cells/well in 6-well plates and cultured in osteoblast differentiation medium containing different concentrations (0–10 uM) of baicalein. Two weeks later, the medium culturing cells were removed and the cells were washed with PBS twice. TDSCs were fixed in 4% paraformaldehyde at room temperature for 30 minutes, alizarin red staining (Sigma-Aldrich) was conducted according to the instructions, and a microscope was used (Olympus) to take pictures to observe these results. Parallel wells of each group were added in an equal amount of sixteen alkyl pyridine chloride solutions to dissolve mineralized nodules. The OD values at 560 nm were measured by a multifunctional microplate reader and then by comparing OD values testing the number of mineralized nodules among the groups.

### 2.9. Real-Time Quantitative PCR (q-PCR)

Total RNA was isolated from TDSCs with the TRIzol reagent (Life Technologies, Grand Island, NY, USA) according to the manufacturer's instructions after 2 weeks of osteogenic induction and the concentration of RNA was detected by NanoDrop 8000 ultraviolet-visible spectrophotometer (Shanghai Fu Ze trade Co., Ltd., China). cDNA Synthesis Reagents Kit (iScript cDNA Synthesis Kit, Bio-Rad company of USA) and Veriti® 96-Well Thermal Cycler instrument were used for reverse transcription. According to the reverse transcription kit, cDNA which was obtained from above reverse transcription added samples and was detected by Q-PCR sequence detection system (7900HT, Applied Biosystems Inc.; USA). Primer sequences (Life Technologies) used in this study are shown in Table 1 [18, 30]. And the data were analyzed by 2^−ΔCt^ method [31].

### 2.10. Western Blot Assay

After culturing with various concentrations of baicalein in osteoblast differentiation medium for 14 days, the osteoblast differentiation protein expression of TDSCs was analyzed with Western blot assay. And its concrete steps and methods were referred to Li et al. (2014) [18]. The primary antibodies used for Western blot analysis were as follows: anti-Runx2 (Cell Signaling Technology, Danvers, MA, USA), anti-OSX (Cell Signaling Technology), anti-OCN (Santa Cruz Biotechnology, Santa Cruz, CA, USA), anti-*β*-actin (Santa Cruz Biotechnology), anti-*β*-catenin (Santa Cruz Biotechnology), and goat anti-rabbit secondary antibody (Santa Cruz Biotechnology). Signals were revealed using an enhanced chemiluminescence kit. The gray values were detected through scanning with Image Pro Plus 6.0 (Media Cybernetics Inc. USA).

### 2.11. Statistical Methods

The data are representative of at least three independent experiments. Each experiment was done in triplicate. The data were graphed using GraphPad Prism software version 3.0 (GraphPad Software Inc., La Jolla, CA, USA). One-way analysis of variance followed by Student's *t*-test was used to determine statistical differences for comparison among groups and independent-sample *t*-test was adopted to determine statistical differences for comparison between groups. Error bars represent the standard error of the mean in the cell experiment and the standard deviation in the animal experiment.

## 3. Results

### 3.1. Baicalein Increases Biomechanical Strength of Tendon-Bone Healing in Rats

**Figure 2:**
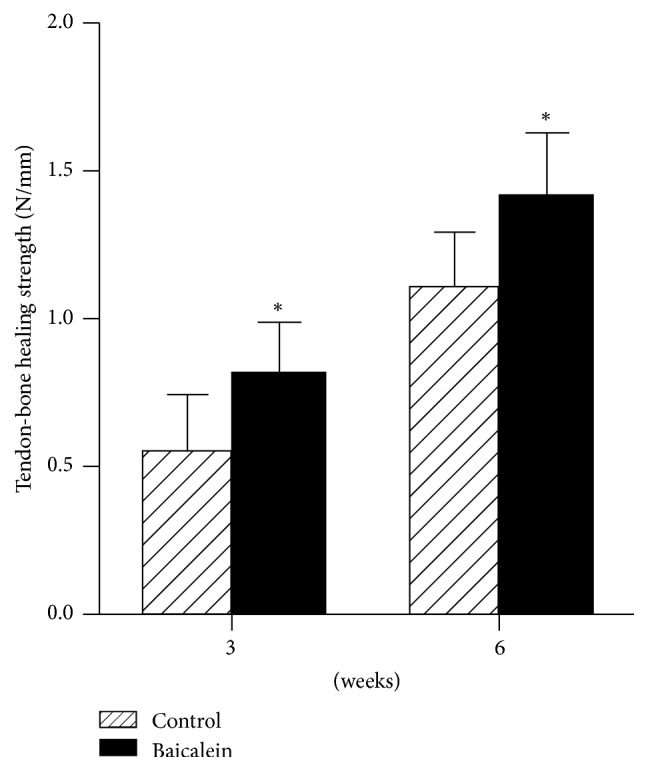
The data shown are the mean ± standard error from independent experiments. ^*∗*^*P* < 0.05 versus group without baicalein with independent-sample *t*-test.

Biomechanical testing was performed at 3 and 6 weeks after the operation. These results showed that tendon-bone healing strength in the experimental group was significantly higher than that in the control group at 3 weeks and 6 weeks after operation, and the difference was statistically significant (*P* < 0.05). (Figure 2)

### 3.2. The Types of Connection between Tendon Graft and Bone Tunnel

At 3 weeks after operation: in control group, tendon-bone interface mainly was loose connective tissue; in the experimental group, the tendon-bone interface also mainly was loose connective tissue. And at 6 weeks after operation: the tendon-bone interface of the control group was mainly composed of loose connective tissue; the interface of the experimental group was mainly dense connective tissue. The details of the connection types between tendon graft and bone tunnel were shown in Table 2. Two groups did not appear to have typical direct insertion [5].

### 3.3. Baicalein Accelerates Osteogenesis of Tendon-Bone Healing Interface

**Figure 3:**
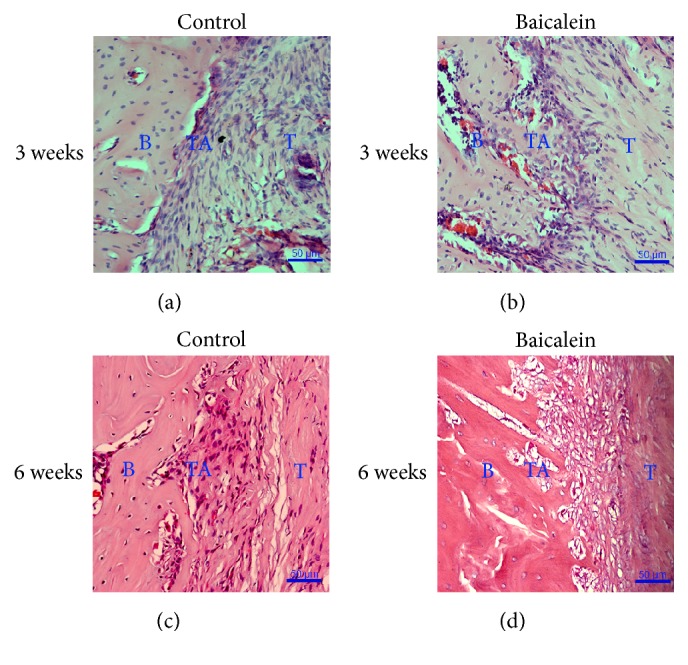
Photomicrographs of HE stained tissue sections (5 um) of the control group ((a) and (c)) and baicalein group ((b) and (d)) at 3 and 6 weeks after surgery (magnification: 200x). B: bone area; TA: transition area; T: tendon area.

At 3 weeks after operation, the tendon-bone interface in the control group was that a large number of fibroblasts were gathered, cells were arranged in disorder with no fixed arrangement, no chondrocyte-like cells were found in the vicinity of the bone tunnel, interstitial calcification was not obvious, and the mass of new bone was less on the surface of the tunnel (Figure 3(a)). In the experimental group, there were a large number of fibroblasts in the tendon-bone interface, the cells were arranged in order, some chondrocyte-like cells existed in the vicinity of the bone tunnel, cells interstitial had the tendency of calcification, and the mass of the new bone on the surface of tunnel was more (Figure 3(b)).

At 6 weeks after operation, the tendon-bone interface in the control group was that there were six cases of Sharpey fibers, cells arrangement was in disorder, chondrocyte-like cells were found near the tunnel wall, stromal cells had calcification tendency, and a little new bone was found on the surface of tunnel wall, but within the tendon-bone interface it had no new bone formation (Figure 3(c)). In the experimental group all tendon-bone interface existed in obvious Sharpey fiber connection, cells arranged in order, the number of chondrocyte-like cells were more near bone tunnel, the matrical calcification was obviously higher than those of the control group, lamellar new bone formed at the tendon-bone interface, and the mass of new bone formation was much more on tunnel wall surface (Figure 3(d)).

### 3.4. Effect of Baicalein on TDSCs Proliferation

**Figure 4:**
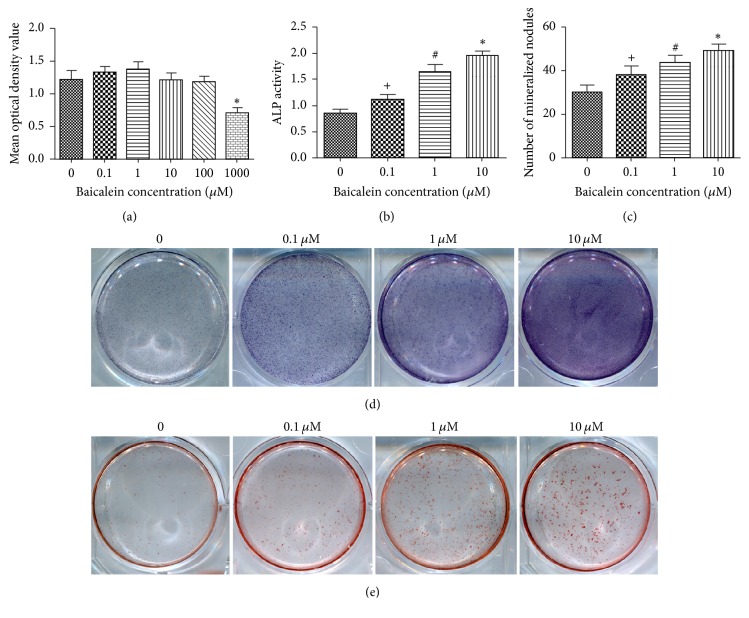
Effect of baicalein on TDSCs proliferation (a); effect of baicalein on TDSCs ALP activity (b) and ALP staining (d); effect of baicalein on TDSCs mineralized nodule count (c) and alizarin red staining (e).* Notes*. (a) The OD values shown are the mean ± standard error of the data from experiment. ^*∗*^*P* < 0.05 versus group without baicalein. (c, e) The results showed that baicalein can increase the number of mineralized nodules (c, e). ^+^*P* < 0.05 versus group without baicalein; ^#^*P* < 0.05 versus group with 0.1 uM baicalein; ^*∗*^*P* < 0.05 versus group with 1 uM baicalein. ALP, alkaline phosphatase; CCK-8, cell counting kit-8 colorimetric assay; TDSCs, tendon-derived stem cells.

The effect of baicalein on TDSCs proliferation was measured by the CCK-8 Kit. The assay results showed that the effect of 0.1, 1, 10, and 100 *μ*M baicalein after treatment for 14 days was no apparent cells toxicity, but 1000 *μ*M of baicalein significantly inhibited cell proliferation and viability (Figure 4(a)).

### 3.5. Baicalein Promotes Osteogenesis of TDSCs* In Vitro*

**Figure 5:**
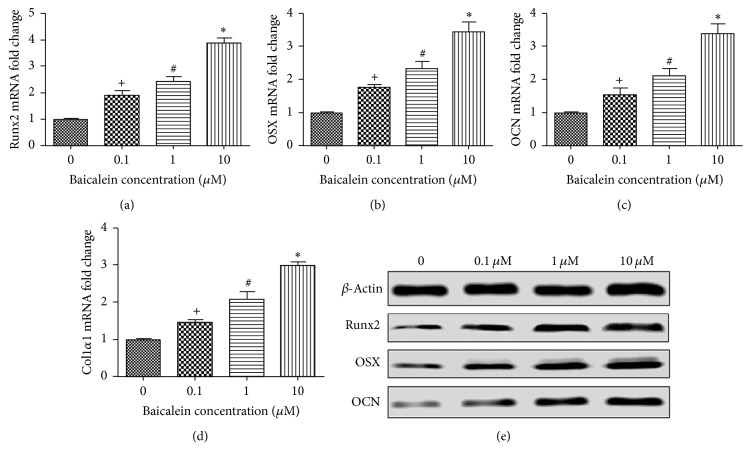
(a–d) Effects of baicalein on gene expression of RunX2, OSX, OCN, and Col1*α*1; (e) effects of baicalein on protein expression of RunX2, OCN, and OSX.* Notes*. Data are expressed as fold change versus the control group, taken as calibrator for comparative quantitation analysis of GAPDH mRNA levels. The values shown are the mean ± standard error of data from independent experiments. ^*∗*^*P* < 0.05 versus group without baicalein; ^+^*P* < 0.05 versus group with 0.1 uM baicalein; ^#^*P* < 0.05 versus group with 1 uM baicalein. RunX2, runt-related transcription factor 2; Col1*α*1, collagen 1*α*1; GAPDH, glyceraldehyde-3-phosphate dehydrogenase; OCN, osteocalcin; TDSCs, tendon-derived stem cells; OSX, osterix.

*In vitro*, ALP staining (Figure 4(d)) showed that baicalein could increase the mass of ALP release and ALP activity detection (Figure 4(b)) showed that baicalein could enhance TDSCs ALP activity. Mineralized nodule count (Figure 4(c)) and alizarin red staining (Figure 4(e)) results showed that baicalein could significantly promote TDSCs osteogenesis. q-PCR (Figures 5(a), 5(b), 5(c), and 5(d)) showed that the different concentrations of baicalein could promote the gene expression of osteogenic factors (Runx2, OCN, OSX, and Col1*α*1) and Western Blot (Figure 5(e)) showed that baicalein could promote the protein expression of osteogenic factors (Runx2, OCN, and OSX). These results also showed that this response was concentration-dependent and peaked at 10 *μ*M. These results suggested that baicalein promotes osteogenesis of TDSCs* in vitro*.

### 3.6. The Effect of Baicalein on Wnt/*β*-Catenin Signaling Pathway

**Figure 6:**
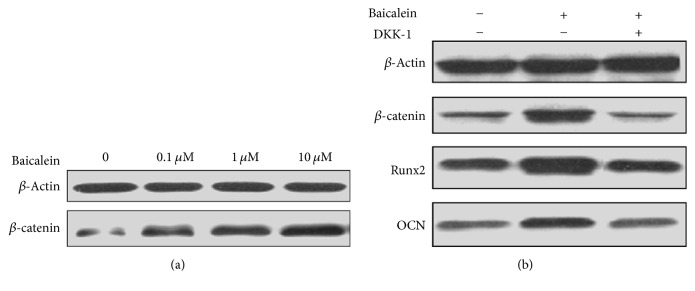
(a) The effect of baicalein on *β*-catenin protein expression. (b) With or without adding DKK-1, the effect of baicalein on the protein expression of *β*-catenin, OCN, and RunX_2_ protein of TDSCs.* Notes*. + indicates adding this kind of test reagent. − indicates that this kind of test reagent is not added. RunX2, runt-related transcription factor 2; OCN, osteocalcin; TDSCs, tendon-derived stem cells; DKK-1, dickkopf-1.

Western blotting results showed that baicalein could promote the *β*-catenin protein expression of TDSCs in a dose-dependent manner and peaked at 10 *μ*M (Figure 6(a)). After adding the inhibitor of Wnt/*β*-catenin signaling pathway DKK-1(Sigma-Aldrich), the effect of baicalein on the promotion of *β*-catenin protein expression and osteogenesis protein (RunX2, OCN) expression could be inhibited (Figure 6(b)).

## 4. Discussion

In this study, we tested the hypothesis that baicalein promotes tendon-bone healing by osteogenic differentiation of TDSCs with a combination of* in vivo* and* in vitro* approaches.* In vivo,* biomechanical testing demonstrated that tendon pulling out strength of the experimental group was significantly higher than that of the control group. These results suggested that bone ingrowth, mineralization, and incorporation of the healing tissue of the baicalein treatment group were stronger than those of the control group [1]. The tendon graft and bone tunnel interface type showed that the experimental group of tendon-bone healing type was better than that of the control group. After treatment of baicalein, the tendon-bone interface collagen grew better, and the Sharpey fiber occurred earlier than that of the control group. At the same time, the Sharpey fiber is regarded as the earliest sign of osteointegration [5, 32]. The cause of the two groups not appearing to have typical direct insertion may be related to the follow-up time of the previous animal studies being relatively short [5]. All of the above results suggested that the effect of baicalein promotes tendon-bone healing in a tendon-bone healing rat model.* In vitro,* TDSCs were regarded as the target to clarify the mechanism of baicalein on tendon-bone healing through the influence of baicalein on TDSCs osteogenic differentiation. After treatment of baicalein, the expression of osteogenic differentiation markers of TDSCs, that is, ALP, mineralized nodule, RunX2, OCN, and OSX of TDSCs, were upregulated. These data suggested that baicalein promotes osteogenesis of TDSCs, and this effect was concentration-dependent.

Wnt/*β*-catenin pathway has been shown to be crucial in bone development and received considerable interest as a potential target [33–35]. In recent years, a large number of studies have reported that Wnt/*β*-catenin signaling pathway plays an important role in osteogenic differentiation and bone formation. These findings demonstrated that the activation of the Wnt/*β*-catenin signaling pathway can induce osteoblastic differentiation, inhibit osteoclast differentiation, and suppress chondrocytic formation [33, 34, 36, 37]. It has demonstrated that stimulating tendon graft in a bone tunnel osseous ingrowth can accelerate the tendon-bone healing progress. The process of tendon graft osseous ingrowth may be activation of Wnt/*β*-catenin signaling pathway. Therefore, baicalein may promote osteogenesis of TDSCs to enhance tendon graft bone ingrowth or tendon-bone healing progress via activation of Wnt/*β*-catenin signaling pathway. In our study, the results showed that baicalein promoted TDSCS osteogenic differentiation and also activated the Wnt/*β*-catenin signaling pathway. And this effect of osteogenic differentiation can be significantly inhibited in the use of DKK-1. Therefore, baicalein promotes the osteogenic differentiation of TDSCs through activation of Wnt/*β*-signaling pathway, which promotes bone formation of the tendon-bone interface to enhance tendon-bone healing progress.

In this study, a new relationship between traditional Chinese medicine monomer and tendon-bone healing was established which offered a new method to achieve tendon-bone healing and the molecular mechanism of tendon-bone healing was clarified. Baicalein extracted from the root of* Scutellaria baicalensis* is easy to obtain and of low cost. Not like permanently implanted polymeric devices, baicalein which is safe and can metabolize in the body does not have the possible long-term adverse effects. And we can regulate the dose of taking baicalein orally after an operation to get the best therapeutic effect.

Unfortunately, we have to point out that there are some limits of our study. The observation time of baicalein on tendon-bone healing* in vivo* was relatively short, and we did not discuss the molecular mechanisms of baicalein on tendon-bone healing* in vivo*. Notwithstanding its limitation, we will future observe the effect of baicalein on tendon-bone healing through prolonging observation time and test the molecular mechanisms* in vivo*.

In conclusion, our study demonstrated that baicalein stimulates osteogenic differentiation of TDSCs via activation of Wnt/*β*-catenin signaling pathway to accelerate tendon-bone healing. Baicalein may have potential application in the treatment of tendon-bone healing.

## Figures and Tables

**Table 1:** Primer sequences for q-PCR.

Target gene	Sequences (5′-3′)
Collagen Ι	F, AGTGGTTTGGATGGTGCCAA; R, GCACCATCATTTCCACGAGC
RUNX2	F, AGTGGTTTGGATGGTGCCAA; R, CACTTCTAGGTCTGACGACG
OCN	F, TCTATGACCTGCAGAGGGCT; R, ATAGCTCGTCACAAGCAGGG
OSX	F, TCTCAAGCACCAATGGACTCCT; R, GGTAGTCATTTGCATAGCCAGA
GAPDH	F, CAGGGCTGCCTTCTCTTGTG; R, GATGGTGATGGGTTTCCCGT

F, forward; R, reverse; OCN, osteocalcin; Runx2, runt-related transcription factor 2; GAPDH, D-glyceraldehyde-3-phosphate; OSX, osterix.

**Table 2:** The types of interfacial morphology.

		Direct insertion	Indirect insertion	Connective tissue	Tendon-bone separation
3 weeks	Control Baicalein	0 0	0 3	7 6	3 1
6 weeks	Control Baicalein	0 0	4 8	6 2	1 0
